# Non-pharmacologic approaches to treatment of pediatric functional abdominal pain disorders

**DOI:** 10.3389/fped.2023.1118874

**Published:** 2023-06-15

**Authors:** Partha Sarathi Chakraborty, Rhea Daniel, Fernando A. Navarro

**Affiliations:** Department of Pediatrics, Division of Gastroenterology, Hepatology and Nutrition, McGovern Medical School, The University of Texas Health Science Center at Houston, Houston, United States

**Keywords:** functional gastrointestinal disorders, functional abdominal pain disorders, irritable bowel syndrome (IBS), non-pharmacologic, treatment

## Abstract

Functional abdominal pain disorders (FAPDs) affect up to 25% of children in the United States. These disorders are more recently known as disorders of “brain-gut” interaction. The diagnosis is based on the ROME IV criteria, and requires the absence of an organic condition to explain the symptoms. Although these disorders are not completely understood, several factors have been involved in the pathophysiology including disordered gut motility, visceral hypersensitivity, allergies, anxiety/stress, gastrointestinal infection/inflammation, as well dysbiosis of the gut microbiome. The pharmacologic and non-pharmacologic treatments for FAPDs are directed to modifying these pathophysiologic mechanisms. This review aims to summarize the non-pharmacologic interventions used in the treatment of FAPDs including dietary modifications, manipulation of the gut microbiome (neutraceuticals, prebiotics, probiotics, synbiotics and fecal microbiota transplant) and psychological interventions that addresses the “brain” component of the brain-gut axis (cognitive behavioral therapy, hypnotherapy, breathing and relaxation techniques). In a survey conducted at a large academic pediatric gastroenterology center, 96% of patients with functional pain disorders reported using at least 1 complementary and alternative medicine treatment to ameliorate symptoms. The paucity of data supporting most of the therapies discussed in this review underscores the need for large randomized controlled trials to assess their efficacy and superiority compared to other treatments.

## Introduction

Functional gastrointestinal disorders (FGIDs) are characterized by recurrent abdominal pain in the absence of structural or biochemical abnormalities (1). The pathophysiology of FGIDs is multifactorial and thought to involve disturbances in gastrointestinal motility, visceral pain perception, intestinal permeability, and gut microbiota. There are no specific biomarkers for diagnosis. Diagnosis is clinical and currently based on Rome IV criteria (2). FGIDs in children can be further classified into three main categories: disorders of nausea and vomiting, disorders of defecation, and pain-based [functional abdominal pain disorders (FAPD)]. FAPD can be further classified into 4 subcategories: irritable bowel syndrome (IBS), abdominal migraine (AM), functional dyspepsia (FD), and functional abdominal pain-not otherwise specified (FAP-NOS). FGIDs may impair quality of life (QoL), social functioning, school attendance, and contribute to increased healthcare costs (3). Pharmacologic and non-pharmacological treatments have been used in the treatment of FGIDs. Data on non-pharmacologic treatments are scarce in the pediatric population and often difficult to interpret due to small sample sizes. Despite the lack of clear evidence for the efficacy of many non-pharmacologic treatments, in a survey conducted at a large academic pediatric gastroenterology center, 96% of patients with FGIDs reported using at least 1 complementary and alternative treatment to ameliorate symptoms (4). Given the widespread patient interest in non-pharmacologic therapies it is important for providers to be aware of these treatment options and the evidence supporting or not supporting their use. This review aims to summarize data available on commonly encountered non-pharmacologic treatment modalities in the management of pediatric FGIDs.

## Dietary changes

### Low fermentable oligosaccharides, disaccharides, monosaccharides and polyols diet (FODMAP)

The low FODMAP diet has been one of the most well studied interventions in FGID management in adults. The low FODMAP diet aims to minimize the intake of several fermentable carbohydrates, including foods containing galacto-oligosaccharides, lactose, fructose in excess of glucose, and sugar polyols, such as sorbitol and mannitol. Examples of foods high in FODMAPs are: wheat, rye, barley, brussels sprouts, artichoke, asparagus, lentils, legumes, okra, peas, apples, shallots, beet, broccoli, fennel and onion (5). The low FODMAP diet is considered an effective dietary approach in patients with IBS especially if symptoms persist after lifestyle changes and other dietary restrictions. Some studies have also shown that FODMAPs can alter gut microbiome and increase visceral nociception (6, 7). Restrictive diets such as the low FODMAP diet needs to be administered under the guidance of a clinician as there is a risk of nutritional inadequacy and development of poor eating behaviors and food fears (8). In pediatrics, 2 well designed studies have been conducted investigating the use of a low FODMAP diet for the treatment of FGIDs (Table 1). The studies reported conflicting results. A randomized double- blind cross over clinical trial by Chumpitazi et al. including 33 children demonstrated a significantly lower number of daily episodes of abdominal pain after 48 hours of a low FODMAP diet compared to children on a typical American diet (9). The study also suggested microbiome composition may play a role in responsiveness to the diet with children who responded having a baseline microbiome composition enriched with taxa known to have greater saccharolytic metabolic capacity (Bacteroides, Ruminococcaceae and Faecalibacterium prausnitzii) than non-responders (9). Another double- blind randomized controlled single center trial conducted in Poland included 27 patients with FAP and found that children randomized to a low FODMAP diet had a trend towards improvement in abdominal symptoms compared to children randomized to the control diet [the control diet was based on the National Institute for Health and Care Excellence guidelines (NICE)] but the difference did not reach statistical significance (10). The lack of efficacy of the FODMAP diet found in this study could be due to the small sample size vs. the control diet used in the study not being reflective of a typical child's diet in the United States. The control diet employed in the study was based on NICE guidelines which are founded in healthy eating principles (eating regular meals, eating slowly, avoiding fast foods, caffeinated beverages, sugar free sweets and sorbitol, and drinking at least 8 cups of fluid per day) (10).

**Table 1:** Low FODMAP diet.

	Sample size	Age in years	Diagnosis	Study design	Conclusion
Chumpitazi et al., 2015 (9)	52 enrolled; 33 completed the study	7–17	IBS diagnosed by ROME III	Randomized double-blind crossover	Significantly lower number of daily episodes of abdominal pain on a low FODMAP diet 1.1 ± 0.2 (SEM) episodes/day vs. 1.7 ± 0.4, *P* < 0.05 compared to children on a typical American diet
Boradyn et al., 2020 (10)	29 enrolled, 27 completed the study	5–12	FAP diagnosed by ROME III (excluded patients with IBS, FD or AM)	Double blind RCT	The low FODMAP diet was not effective in reducing of symptoms of FAP

### Lactose restricted diet

Lactose is a disaccharide comprised of glucose and galactose. Lactose is digested by hydrolysis via lactase-phlorizin hydrolase (LPH) activity in the small intestinal brush border. Symptoms of lactose intolerance (diarrhea, bloating, and abdominal pain) resulting from LPH deficiency often overlap with those seen in IBS (11). Studies have not found an increased prevalence of LPH deficiency in patients with IBS compared to the general population (12, 13). The role of lactose elimination in the management of IBS is controversial and some recommend obtaining a lactose breath test before recommending a lactose free diet to prevent unnecessary dietary restrictions in lactase persistent IBS patients (14). Although lactose breath testing or lactase determination obtained from intestinal biopsies may help provide evidence for or against lactose restriction, a finding of lactase deficiency may not be the underlying cause of symptoms in patients with IBS (Table 2). A double-blind cross over design study by Gremse et al. included 30 children with IBS and a diagnosis of lactose maldigestion made by lactose breath hydrogen testing and found that 23 of 30 patients reported more pain when ingesting lactose containing milk (15). Although most children did have increased symptoms with the ingestion of lactose, 23% either reported no abdominal pain or less symptoms with the ingestion of lactose suggesting some of the symptoms attributed to lactose indigestion may be secondary to another condition such as IBS (15). Another double-blind placebo-controlled provocation study with similar results conducted by Gijsbers et. al found that in 220 children with recurrent abdominal pain, 27% had abnormal lactose hydrogen breath tests indicative of lactose maldigestion but no causal relationship could be established (16). In this study, causal relationship between lactose and symptoms was defined as a disappearance of abdominal pain with elimination, recurrence with provocation, and disappearance with re-elimination followed by a pain-free period of at least 6 months. In a study conducted by Lebenthal et al. after a 12-month milk elimination diet, 40% of children with lactose malabsorption and 38% of lactose tolerant children reported elimination of their pain (17). 42% of children with recurrent abdominal pain who received a regular diet for 1 year had improvement in their symptoms. These results suggests dietary elimination of lactose does not affect the overall frequency of improvement (17).

**Table 2:** Lactose restricted diet.

	Sample size	Age in years	Diagnosis	Study design	Outcome
Gremse et al., 2003 (15)	30	3–17	Recurrent abdominal pain of childhood and lactose maldigestion diagnosed by abnormal breath hydrogen test	Randomized double-blind crossover	7/30 subjects reported less pain or no change in pain with lactose ingestion. Symptom scores were similar for subjects with a 10–20 ppm increase in breath hydrogen and those with a > 20 ppm increase in breath hydrogen. Ingestion of lactose is associated with increased abdominal pain in susceptible children with lactose maldigestion
Gijsbers et al., 2012 (16)	220	4.1–16	Recurrent abdominal pain	Double blind placebo-controlled provocation	No causal relationship between lactose or fructose and recurrent abdominal pain could be established
Lebenthal et al., 1981 (17)	103	6–14	Recurrent abdominal pain	3 successive 6-week diet trials completed in a double-blind fashion (*n* = 38); 40 patients followed for 12 months either on lactose free (*n* = 28) or lactose containing diet (*n* = 12)	The prevalence of lactase deficiency was similar between controls and subjects with recurrent abdominal pain. At 12 months, lactose elimination had no effect on outcomes for lactose absorbers vs. malabsorbers

### Fiber supplementation

A meta-analysis conducted in 2015 included 22 randomized controlled trials (RCTs) with 1,299 total participants ranging in age from 15 to 78 years old and found soluble fiber improved global symptom scores in patients with IBS while insoluble fiber did not appear to have an effect (18). Although this meta-analysis included some studies with pediatric patients, the majority of subjects were adults. Studies conducted exclusively in pediatric populations have shown some conflicting results but most support the use of soluble fiber (Table 3). One double-blind RCT including 84 children found that the administration of soluble fiber in the form of glucomannan twice a day for 4 weeks (total dose of 2.52 grams of fiber per day) did not provide any benefit compared to placebo (19). In contrast, a double blind RCT including 103 children found that although the level of pain intensity did not differ between groups, children randomized to receive psyllium (a form of soluble fiber) for 6 weeks had greater reductions in mean number of pain episodes compared to the placebo group (20). The differences in findings between these two studies may be the smaller sample size and uniform fiber dosing regardless of weight or age in the former vs. larger sample size and fiber dosing based on age in the latter study. Menon et al. conducted a randomized double blind placebo controlled superiority trial (*n* = 81) and also found children with FAPD receiving age based doses of soluble fiber (psyllium) for 4 weeks reported reduced IBS symptoms compared to the placebo group (21). Similarly, a double blind RCT in 60 children by Romano et al. found partially hydrolyzed guar gum (a form of soluble fiber) did significantly reduce clinical symptoms in subjects with chronic abdominal pain (22).When counseling patients on fiber supplementation, a gradual increase in daily fiber intake is recommended to avoid adverse symptoms such as bloating and flatulence. Soluble fiber can be found in psyllium, ispaghula husk, corn fiber, calcium polycarbophil, methylcellulose, oat bran, and the flesh of fruits and vegetables while insoluble fiber is found in wheat bran, whole grains, fruit and vegetable skins, and seeds (23). The mechanism of action of dietary fiber is not clear but it is dependent on the type of fiber consumed. Dietary fiber can be divided into different types including soluble (viscous or non-viscous) or non-soluble as well as short chain vs. long chain carbohydrates and fermentable or non-fermentable (24). The dietary fiber that is associated with improvement in IBS symptoms is soluble, viscous, and moderately fermented. Some proposed mechanisms for how fiber works to improve symptoms in IBS include a laxative effect of fiber where soluble fiber fermented by colonic bacteria increases stool bulk through increased biomass production via fermentation by-products (24). Soluble viscous fibers form a gel as they pass through the colon and the gel is excreted in stool which may improve stool texture (24). Short chain fatty acid (SCFA) by-products of fiber fermentation (primarily acetate, propionate, and butyrate) alter the luminal colonic pH promoting growth of beneficial bacterial species (24). SCFAs have been studied in the pathophysiology of IBS with research in adults showing that compared to controls, patients with constipation predominant IBS have lower levels of SCFAs and those with diarrhea predominant IBS have increased levels (25). SCFAs, particularly butyrate, has been implicated in multiple pathways including the intestinal neuroendocrine system, inflammatory response regulation, intestinal barrier integrity, and colonic motility (25).

**Table 3:** Fiber supplementation.

	Sample size	Age in years	Diagnosis	Study design	Outcome
Horvath et al., 2013 (19)	89	7–17	Abdominal pain related FGID diagnosed by ROME III criteria	Double blind placebo controlled randomized trial	Glucomannan administered twice daily (2.52 g/day) for 4 weeks had no benefit compared to placebo in reducing the severity or frequency of pain
Shulman et al., 2017 (20)	103	10–16	IBS diagnosed by ROME III criteria	Double blind RCT	After 6 weeks, children randomized to receive psyllium had greater reductions in mean number of pain episodes compared to the placebo group
Menon et al., 2023 (21)	81	4–18	IBS diagnosed by ROME IV criteria	Double blind RCT	After 4 weeks, children randomized to receive psyllium had greater reductions IBS symptom severity scores than the placebo group and 43.9% of children in the psyllium group were in remission at the end of treatment compared to 9.7% in the placebo group
Romano et al., 2013 (22)	60	8–16	FGID diagnosed by ROME III criteria	Double blind RCT	Children randomized to the partially hydrolyzed guar gum group had significantly reduced mean scores in evaluation of principal IBS related symptoms compared to the placebo group at both 4 and 8 weeks

### Gluten free diet

Non-celiac gluten sensitivity (NCGS) is characterized by intestinal and or extra intestinal symptoms after gluten ingestion that resolve with gluten withdrawal and relapse following a gluten challenge in the absence of celiac disease (CD) or wheat allergy (26). Intestinal symptoms of NCGS tend to be IBS-like in nature and a gluten-free diet (GFD) has been investigated for treatment of IBS. In pediatrics, the prevalence of NCGS and efficacy of gluten avoidance in symptom management is not well studied. One study conducted at an Italian high school showed that of 555 students 12.2% self-reported NCGS (27). 68% of the students who self-reported NCGS were female and 2.9% of students were adhering to a GFD (27). Another study included 1,114 children with FGIDs and found 3.3% of subjects correlated symptoms with gluten ingestion (28). Those subjects (*n* = 36) entered a prospective double-blind placebo-controlled gluten challenge (the study included 3 phases: run-in, open GFD, and double-blind placebo controlled cross over gluten challenge) and results showed only 14% of children had true NCGS (28). This study had 28 subjects complete the prospective arm of the study and included children with IBS as well as other FGIDs (28). Currently, there are no other gold-standard studies (double-blind placebo-controlled gluten challenge with cross-over) investigating NCGS in children (Table 4).

**Table 4:** Gluten free diet.

	Sample size	Age in years	Diagnosis	Type of study	Outcome
Francavilla et al., 2018 (28)	1,114	11.4 ± 4.3	NCGS	Double blind randomized placebo controlled cross over trial	Out of 1,114 subjects with chronic gastrointestinal symptoms, 3.3% correlated their symptoms with gluten. Of those subjects (*n* = 36) 14% were found to have true NCGS

## Modulation of the microbiome

### Probiotics

Probiotics are live microorganisms, that when given in adequate amounts, confer a health benefit to the host (29). Probiotics have been found to be beneficial in infectious as well as antibiotic associated diarrhea, pouchitis, ulcerative colitis, and celiac disease (30–34). Changes in gut microbiota (i.e., intestinal dysbiosis) have been reported in pediatric patients with FGIDs (Table 5). A study conducted by Saulnier et al. analyzed 71 stools samples from 22 children with IBS and 22 healthy controls (participants aged 7–12 years old) and found the microbiota of children with IBS had a greater abundance of Proteobacteria, Veillonella, Prevotella, Lactobacillus, and Parasporobacterium and less of Bifidobacterium and Verrucomicrobium (specifically in children with IBS-diarrhea) (35). A prospective longitudinal study in children between the ages of 7–16 years with (*n* = 28) and without FAP (*n* = 58) found Bacteroidetes significantly lower in the healthy controls and Firmicutes significantly increased in healthy controls (36). Although these studies have a small number of patients, the results are similar to a recent meta-analysis that included 22 studies (3 pediatric studies and 19 adult studies) (37). It has been hypothesized that dysbiosis affects visceral sensitivity, gut motility and intestinal gas production contributing to pain in FGIDs (38). Microbial variations affect epithelial permeability which can induce inflammation by inciting local and systemic responses (39, 40). *Lactobacillus paracasei* NCC2461 and *Lactobacillus acidophilus* NCFM have been found to reduce visceral pain in mice (41). A 2013 meta-analysis by Korterink et al. investigating the use of probiotics in treating childhood FGIDs included 8 studies with pediatric patients between the ages of 2–18 years old and found *Limosilactobacillus reuteri* DSM 17938 (*L. reuteri*) and VSL#3 significantly increased treatment success and reduced pain intensity in children with abdominal pain related FGIDs, especially IBS (42). Despite the effectiveness of *L. reuteri* and VSL#3 in reducing pain related IBS symptoms, probiotics were not more effective than placebo in treating defecation related functional disorders (42). A more recent meta-analysis published in 2020 also examined the use of probiotics and focused specifically on 2 different strains: Lactobacillus rhamnosus (LGG) and *L. reuteri* in the treatment of FGIDs. The meta-analysis included 8 RCTs (3 of which were included in the previously mentioned 2013 meta-analysis) with a total of 641 subjects between the ages of 4–18 years and found a moderate positive effect on pain intensity reduction for *L. reuteri* at 4 weeks however, beyond that there was no significant reduction in pain frequency at any point in time, no reduction in school absenteeism, and no significant increase in the number of children whose symptoms completely resolved after the intervention (43). For LGG, there was no significant effect on any of the outcomes (43). The authors noted that there was a large diversity in outcome reporting, intervention time (ranging from 4 to 12 weeks) and limited number of patients in some studies making them difficult to interpret and compare (43). Although there is still some uncertainty in the efficacy of probiotics in treating FGIDs, there may be some benefit in the use of VSL#3 or *L. reutreri* for reducing pain intensity in patients with IBS and given the good safety profile and low adverse effects, probiotics may be a useful tool for some patients. Evidence of microbial dysbiosis has also been reported in infants with colic compared to infants without colic (44). Infants with colic have been reported to be have less frequent colonization with Lactobacilus species and a greater abundance of anaerobic gram-negative bacteria as well as Clostridium difficile, Escherichia spp and Klebsiella spp, which are known gas producing microorganisms (45, 46). A double blind RCT including 46 infants showed daily use of the probiotic *L. reutreri* for 21 days reduced daily crying time in infants with colic by 50% (47). The results of this study were reproduced in 2 other double blind RCTs (48, 49). Another study also showed that in addition to a significant reduction in infant crying time, the use of *L. reuteri* was associated with decreased maternal depression (50).

**Table 5:** Probiotics and synbiotics.

	Sample size	Age in years	Diagnosis	Study design	Outcome
Saulnier et al., 2011 (35)	44	7–12 years	IBS diagnosed by ROME III criteria	Prospective observational	Patients with IBS had significantly greater percentage of the class *γ*-proteobacteria compared to healthy controls. Several taxa of the genus Alistipes were associated with the phenotype of frequently recurrent abdominal pain
Abomoelak et a. 2021 (36)	28	7–16 years	Functional abdominal pain disorders	Prospective longitudinal study	The phylum Bacteroidetes was significantly higher in subjects with functional abdominal pain vs. controls and Firmicutes significantly increased in healthy controls
Gholizadeh et al., 2021 (51)	67	4–15 years	FAP	Double blind placebo controlled randomized trial	A synbiotic composed of fructo-oligosaccharides and seven types of beneficial bacteria was significantly superior to placebo in decreasing the duration, frequency, and intensity of abdominal pain

Synbiotics are a combination of probiotics and prebiotics (non-digestible foods which benefit the host by stimulating selective bacterial growth). A study by Gholizade et al. published in 2021 demonstrated that a synbiotic composed of fructo-oligosaccharides and seven types of beneficial bacteria effectively decreased the frequency, duration and intensity of FAPD in children (51).

## Psychosocial interventions

Studies have shown that children with FGIDs have a greater incidence of clinically proven anxiety disorders and depression compared to children without FGIDs (52). These psychological symptoms have been associated with visceral hyperalgesia and abnormal gut motility (53). A study by Walker et al. demonstrated that children with chronic abdominal pain had less confidence in their ability to change or adapt to stress and had inferior coping strategies compared to healthy controls (54). Many studies have been conducted investigating various psychosocial interventions aimed at reducing anxiety and depression symptoms and teaching coping strategies (55). Some of the more common psychosocial interventions including interoceptive cognitive behavioral therapy (CBT), gut directed hypnotherapy (HT), and yoga therapy are discussed in more detail below.

### CBT

CBT focuses on reducing anxiety and stress related to somatic sensations (56). Visceral sensitivity and symptom hypervigilance has been recognized as characteristics frequently found in patients with IBS (57, 58). CBT, with a focus on gastrointestinal symptoms, (symptom replication during sessions are achieved via various methods i.e., tightening abdominal muscles, wearing an abdominal compress, eating foods that are avoided, etc.) has been employed as a form of treatment in patients with IBS (Table 6). A 2017 Cochrane review investigating the use of CBT in the treatment of pediatric FGIDs combined 4 RCTs with a total of 175 subjects and found CBT to be effective despite the clinical heterogeneity and small number of patients (55). A small pilot study (*n* = 24) conducted by Zucker et al. showed that CBT can be effectively utilized in younger children (ages 5–9 years) with a focus on promoting playful and curious approaches to body signals and providing families with decision trees to help describe, label, and interpret the meaning of somatic signals through the use of cartoon characters as metaphors for experience (i.e., Gassy Gus) (59). Although studies have shown CBT may be efficacious in the treatment of IBS, accessibility to these services is a barrier to care. Internet and telephone-based methods for administering CBT have been investigated as a potential way to increase access to care. A RCT conducted in 101 adolescents (13–17 years of age) examined the effectiveness of internet delivered CBT for IBS treatment and found internet-based CBT to be more effective than the wait-list control group in reducing the severity of overall gastrointestinal symptoms, pain intensity, pain frequency, avoidant behavior, fear of symptoms, medication use, school absenteeism and QoL with a number needed to treat of 4 (60). CBT targeted at parents alone has also been studied in the treatment of pediatric FGIDs. A prospective longitudinal RCT conducted by Levy et al. included 316 parent child dyads (children aged 7–12 years) and found CBT delivered to parents by phone or in-person for 3 sessions resulted in no significant difference in reported pain severity or symptoms by children however, secondary outcomes of parental catastrophizing, parent-reported child disability, QoL, and utilization of healthcare for pain did show significant improvement compared to controls (61). It is also notable that in this study CBT delivered via phone or in-person had comparable results with no significant difference between the two groups (61). Parents who were more likely to complete the study were more likely to be older in age and married (61). Another limitation to successful treatment with CBT includes patient motivation and engagement in therapy sessions. A study in adults examining individual characteristics that would identify patients more or less likely to respond to CBT was not able to identify any reliable predictors (62).

**Table 6:** Interoceptive cognitive behavioral therapy.

	Sample size	Age	Diagnosis	Study design	Outcome
Bonnert et al., 2017 (60)	101	13–17 years	IBS diagnosed by ROME III criteria	RCT	Internet based CBT was more effective than a wait-list control in reducing the severity of overall GI symptoms, pain intensity, QoL, school absenteeism, avoidant behavior, medication use, and fear and worry about symptoms with a number needed to treat of 4.06
Levy et al., 2017 (61)	316	7–12	FGID diagnosed by ROME III criteria	RCT	No significant difference in child reported pain severity or other GI symptoms. Parents reported less health care utilization and school absenteeism
Abott et al. (55)	175	<18	Recurrent abdominal pain	Meta-analysis	CBT is effective despite clinical heterogeneity and small number of patients
Zucker et al. (59)	24 parent-child dyads	5–9	Functional abdominal pain disorders	Prospective longitudinal study	Pain and negative affect demonstrated statistically significant change

### HT

HT has been used for treatment of refractory IBS since the 1980s (63). The aim of (HT) is to induce a deep state of relaxation in order to guide patients in understanding how to control their gut function (63). Sessions are typically conducted in weekly 30–60 minute sessions over 3 months (63). Effectiveness has been demonstrated in multiple trials (Table 7). A 2017 Cochrane review included 4 studies (146 patients across all studies) examining HT or guided imagery in the treatment of pediatric FGID and concluded that for HT or guided imagery compared to control groups, there is evidence of superiority for HT/guided imagery in the outcomes of reduction in pain intensity, and reduction in pain frequency (55). The evidence was rated as low quality due to the common issues of small sample size and risk for bias in the included studies (55). Since the publication of the 2017 Cochrane review, additional pediatric studies investigating HT have been published. One RCT compared the effectiveness of HT vs. standard medical care in 100 children aged 8–18 years of age with functional nausea or FD (64). Children randomized to the HT group received 6 sessions lasting 50–60 minutes over a 3-month time period. Results of the study showed the severity, incidence, and frequency of nausea decreased significantly across both groups but at the 6 month follow up period the HT group had significantly lower mean scores of nausea severity and frequency compared to the control group (64). For patients with functional nausea vs. FD, HT was more effective than standard medical therapy in the first 6 months of treatment compared to controls (40% vs. 13%) (64). Given difficulties with accessibility to therapists able to provide HT services, studies have investigated the effectiveness of non-individualized HT in IBS treatment. A non-inferiority RCT including 250 children found that home based hypnotic treatment [subjects were provided a compact disk (CD) that contained 5 standard scripts of HT exercises] was non-inferior to in-person HT sessions 1 year after the end of treatment (65). In the study, 62.1% of children in the CD group vs. 71% of children in the in-person HT group were successfully treated (65). How HT works to improve symptoms of FAP is not clear. In a RCT that included 46 children with either FAP or IBS, no changes in rectal hypersensitivity (measured by rectal barostat testing) were found after 12 weeks of HT vs. standard medical care (66). This study also questioned the role of rectal hypersensitivity in the pathogenesis of pediatric FAP or IBS as the authors did not find an association between rectal barostat results and clinical symptoms (66). Although the mechanism behind HT is largely unknown, the benefits of HT have been well demonstrated and should be considered in a comprehensive treatment plan for refractory IBS.

**Table 7:** Hypnotherapy.

	Sample size	Age in years	Diagnosis	Study design	Outcome
Browne et al., 2022 (64)	100	8–18	Functional nausea or FD diagnosed by ROME IV criteria	RCT	For patients with functional nausea vs. FD, HT was more effective than standard medical therapy in the first 6 months of treatment compared to controls (40% vs. 13%)
Rutten et al., 2017 (65)	260	8–18	FAP	Non-inferiority RCT	3 months of home based HT was non inferior to 3 months of individualized HT at 1 year after completion of treatment
Abott et al. (55)	146	<18	Functional abdominal pain disorders	Meta-analysis	Post intervention reduction in pain intensity and pain frequency

### Yoga therapy

Yoga incorporates aspects of exercise, breath work, concentration, and meditation (67). Health benefits include improvements in physical and mental health through down regulation of the hypothalamic-pituitary-axis and sympathetic nervous system with multiple studies reporting a beneficial effect on salivary cortisol, blood glucose, heart rate, and blood pressure (67). A 2017 Cochrane review investigating the effectiveness of yoga in the treatment of pediatric FGIDs included 3 studies with a total of 122 children and found no evidence of an effect of yoga therapy on pain intensity immediately post intervention (55). One of the 3 studies included in the review found that although there was no significant difference in the immediate overall treatment success between the usual care plus yoga intervention group vs. usual care group there was a significantly higher treatment success rate at the 12 month follow up (58.1% in the usual care plus yoga group vs. 28.9% in the usual care group). Some barriers to yoga therapy include purchasing equipment needed for different yoga poses and ensuring correct positioning and postures in order to prevent injuries which may make regular home practice difficult. A study conducted by Evans et al. aimed to explore differences between adolescent patients with IBS who responded to yoga therapy vs. those who did not respond (68). The study included 18 participants (ages 14–17 years old) and found 50% responded to yoga therapy (68). Patients who responded reported generalizable benefits after a few sessions while non-responders noted they were just starting to note a response late into the intervention time period (68). Patients who responded also had more parental support with parents not expressing frustration with the time commitment required to drop patients off at yoga practice and having more knowledge of the child's home practice materials compared to non-responders (68). Results of the study suggest some patients may require longer treatment periods and parental buy-in is a key component of success. Yoga may appear to have some benefit in treating IBS symptoms (Table 8), however there is not sufficient evidence available to recommend yoga as treatment for IBS. Given the benefits of exercise on physical and mental health, regular exercise should always be promoted.

**Table 8:** Yoga.

	Sample size	Age in years	Diagnosis	Study design	Outcome
Evans et al., 2018 (68)	18	14–17	IBS diagnosed by ROME III criteria or recurrent abdominal pain	Mixed methods study	50% of patients had a clinically meaningful reduction in pain after 6 weeks of Iyengar yoga
Abott et al. (55)	122	>18	Functional abdominal pain disorders	Meta-analysis	No evidence of post intervention pain intensity reduction

### Other therapies

Principles of acupuncture are based on theories that contemplation and reflection on sensory perceptions and ordinary appearances are adequate to understand health and illness which is contrary to traditional biomedical viewpoints which emphasize objectiveness and quantitative measurements (69). A report by Yang et al. examining the efficacy and safety of acupuncture in children analyzed 24 systematic reviews (*n* = 12,787) but found only one fifth of the reviews were high quality (70). Reviews that were rated as high quality had neutral or negative results (70). 5 conditions including cerebral palsy, nocturnal enuresis, tic disorder, amblyopia, and pain reduction seemed to respond more favorably to acupuncture. However, the efficacy of acupuncture in nausea and vomiting was controversial (70). A 2017 study examining various RCTs conducted to assess the efficacy of acupuncture in the treatment of pediatric pain found acupuncture treatment to be effective in the treatment of procedural pain, infantile colic, pelvic pain, and headaches but evidence was very limited (71). Procedural pain is acute and different in nature from chronic pain experienced by patients with FGIDs. Although acupuncture may show promising results in treatment of pain, it may not treat the pain associated with FGIDs and it is important to note the distinction between different types of pain. A retrospective chart review investigating the use of acupuncture and integrative medicine for the treatment of FD and refractory GERD was done in 6 children. 80% of children reported a decrease in nausea and 75% reported a decrease in pain and all patients were able to be weaned off of proton pump inhibitors (72). Results of this study are difficult to interpret due to the small sample size and the multiple complementary alternative medical treatments received concurrently with acupuncture (72). Overall, the body of evidence for the use of acupuncture in the treatment of FGIDs is lacking. Similarly, studies showing the benefits of reiki, chiropractic care, moxibustion, or reflexology in pediatric FGIDs is scarce.

## Neutraceuticals

### Peppermint oil

Mint plants have been used medicinally for centuries to treat a myriad of human maladies including hirsutism, memory loss, respiratory problems, nausea, and reflux (73). Steam distillation of fresh peppermint leaves yields peppermint oil which contains the pharmacologically active ingredient of menthol (74). Multiple mechanisms for the therapeutic effects of peppermint oil have been described. One of its primary mechanisms of action involves intestinal smooth muscle relaxation by acting as a calcium channel antagonist which impedes muscle contractility (75). Studies have also suggested peppermint oil may exert its therapeutic actions by directly effecting the enteric nervous system through induction of membrane potential depolarization, modulating visceral sensation by acting on specific superfamilies of cation channels located in the gut, and decreasing inflammation by suppression of pro-inflammatory mediators produced by human monocytes (76, 77). A 2022 RCT investigating the effect of peppermint oil on the gut microbiome studied 30 children between 7 and 12 years of age with FAP and randomized them to received different doses of peppermint oil (180 mg, 360 mg or 540 mg of enteric coated peppermint oil per day) (78). Stool samples were obtained at baseline and at the end of 1 week of treatment (78). Results showed no significant difference in overall microbiome composition before and after treatment with peppermint oil (78). Results did show the specific abundance of *Collinsella* was different significantly before and after treatment (lower at baseline) and the Firmicutes to Bacteriodetes ratio was lower in children who received 540 mg of peppermint oil compared to other groups (78). This is currently the only study investigating microbiome changes in children using peppermint oil for treatment of FAPDs. Different doses of peppermint or a longer period of follow up may yield different results. A double-blind randomized placebo- controlled trial investigating the efficacy of peppermint oil studied 42 children between the ages of 8–17 years diagnosed with IBS. The study found after a 2-week trial the children randomized to receive peppermint oil three times daily had significant improvement in severity of pain symptoms compared to placebo (76% vs. 19% respectively) (79). There were no significant changes between the treatment and placebo group regarding symptoms of abdominal rumbling, abdominal distension, belching, gas, or heartburn (79). Another RCT conducted in pediatric patients compared the efficacy of peppermint oil vs. a synbiotic in decreasing duration, severity, and frequency of pain. The study included 88 patients between the ages of 4–13 years of age and found the peppermint oil group had significantly greater improvements in pain duration, frequency, and severity compared to the placebo group as shown in Table 9 (80). The study also found peppermint oil was superior to the synbiotic in decreasing pain duration and severity (80). Peppermint oil is an ingredient also used in STW-5, a liquid preparation comprised of extracts from 9 different herbs (5% of STW-5 comes from peppermint leaves) (81). STW-5 is approved in several European countries for the treatment of IBS and FD (81). A prospective non-interventional study followed 980 children with FGIDs between the ages of 3–14 years old who were treated for 1 week with 10–20 drops of STW-5 (82). Results showed mean upper and lower gastrointestinal symptoms were reduced (mean score 16.1 ± 8.3 at the start of the study and mean score 3.8 ± 4.24 at the end of the study). 38.6% of participants reported symptoms entirely resolved by the end of the treatment period, and school absenteeism was reduced from 67% to 36.1% (82). The study was published as an abstract and did not report *p*-values. It is difficult to draw conclusion from the study due to the short duration of intervention and follow up and lack of comparison group. Although there is some evidence for STW-5 in adult FGIDs, there are no well-designed prospective trials investigating the efficacy of STW-5 in the pediatric population (83). Peppermint oil is generally well tolerated. However, at higher doses adverse effects may be seen. Some patients with hiatal hernias and or reflux may experience worsening of symptoms because peppermint oil may decrease the lower gastroesophageal sphincter tone. At very high doses, peppermint oil has been associated with interstitial nephritis and renal failure (84).

**Table 9:** Peppermint oil.

	Sample size	Age in years	Diagnosis	Study design	Outcome
Kline et al., 2001 (79)	50 patients enrolled, 42 completed the study	8–17	IBS diagnosed by Manning or ROME criteria	Double blind RCT	A pH dependent enteric coated peppermint oil capsule reduced severity of pain but not total gastrointestinal symptom rating scale score or other IBS symptoms
Asgarshirazi et al., 2015 (80)	88	4–13	FGID diagnosed by ROME III criteria	Double blind placebo controlled randomized trial	Peppermint oil was superior to synbiotic in decreasing pain frequency, duration and severity
Thapa et al., 2022 (78)	30	7–12	FAP diagnosed by ROME III criteria	RCT	Overall gut microbiome composition did not differ significantly after treatment with peppermint oil. Firmicutes/Bacteroidetes ratio was lower in children treated with 540 mg of peppermint compared to other doses

### Fennel

Foeniculum vulgare, also known as fennel, is another medicinal herb that has shown promise in multiple studies in adults with IBS (85, 86). It is often studied along with other herbal interventions such as turmeric. Fennel can act as an anti-spasmodic, anti-oxidant and an anti-inflammatory agent (87). Fennel preparations have been shown to decrease crying episodes in infants with colic in multiple studies (88). However, studies assessing the efficacy of fennel preparations in pediatric FGIDs are lacking (88).

### Serum-derived bovine immunoglobulin/ protein isolate (SBI)

SBI is a specially formulated prescription medical food containing >90% protein, of which >50% is immunoglobulin G (IgG) (89). Studies have shown SBI helps improve gastrointestinal symptoms like abdominal discomfort, bloating, urgency, and chronic loose stools in patients with IBS-diarrhea and human immunodeficiency virus-associated enteropathy (90, 91). The proteins contained in SBI directly alter the permeability of the intestinal barrier (92). They also prevent antigen translocation across damaged tight junctions through direct binding and steric hindrance and impact tight junction protein expression by the epithelium (92). SBI has been shown to maintain immune balance in the gut epithelium (92). A small randomized double blind placebo controlled trial investigating the use of SBI for the treatment of diarrhea predominant IBS enrolled 15 subjects (8–18 years of age) and found after 3 weeks of treatment, the intervention group had a significant reduction in abdominal pain, improvement in stool form, and improvement in overall functional disability index and QoL scores compared to the placebo group (Table 10). However, there was no significant difference in the primary endpoint of reduction in stool frequency at the end of the study (93).

**Table 10:** Serum-derived bovine immunoglobulin/protein isolate (SBI).

	Sample size	Age in years	Diagnosis	Study design	Outcome
Arrouk et al., 2014 (93)	15	8–18	Diarrhea predominant IBS diagnosed by ROME III criteria	Double blind RCT	Subjects randomized to the SBI group had improvement in stool form and abdominal pain at 3 weeks.

### Vitamin D

A study investigating the prevalence of vitamin D insufficiency in a group of healthy adolescents (*n* = 307) living in Boston found 42% of participants were vitamin D insufficient with 24% of the study population having vitamin D levels in the deficient range (94). Associations between vitamin D deficiency and IBS have been made in a few studies. A retrospective case control study characterizing vitamin D levels in pediatric patients with IBS reviewed the charts of 116 control subjects and 55 subjects with IBS and found only 7% of subjects with IBS met criteria for vitamin D sufficiency compared to 25% of controls as shown in Table 11 (95). No other studies examining the relationship between vitamin D status in patients with IBS and healthy controls in pediatrics has been conducted. Adult studies have shown similar results where patients with IBS are more likely to have vitamin D insufficiency compared to control groups (96, 97). Associations between vitamin D and IBS have led to studies investigating the use of vitamin D supplementation as a potential treatment option for IBS. Postulated mechanisms for the therapeutic action of vitamin D include modulation of serotonin via interactions between vitamin D and tryptophan-hydroxylase-1(the rate limiting enzyme in serotonin production), restoring intestinal dysbiosis associated with vitamin D deficiency, and improving intestinal barrier function (98–100). One RCT looking at vitamin D supplementation in adolescents 14–18 years old with IBS (*n* = 112) found patients who received 6 months of vitamin D supplementation had significant improvement in IBS symptoms and QoL scores compared to the placebo group (101). This is the only RCT investigating the use of vitamin D in the treatment of IBS in pediatrics. A recent meta-analysis which included 685 patients across 8 RCTs showed patients receiving vitamin D supplements had significant improvement in their IBS symptom severity outcomes however, after excluding 5 out of the 8 trials at moderate to high risk for bias the remaining studies did not show a benefit of vitamin D supplementation (102). Similarly, there was no improvement in IBS QoL scores (102). It is notable that 6/8 studies included in the meta-analysis were conducted in the Middle East limiting the generalizability of findings (102). The meta-analysis did include the 1 pediatric study referenced above however, all the other studies were conducted with adult participants. Although there have been studies demonstrating a higher incidence of vitamin D deficiency in patients with IBS compared to healthy controls studies have not proven vitamin D supplementation to be efficacious in ameliorating IBS symptoms or improving quality of life and should not be routinely recommended for treatment of IBS; however, given the high prevalence of vitamin D deficiency in the general population and increased incidence in those with IBS, screening for vitamin D deficiency and supplementation for general health purposes is reasonable.

**Table 11:** Vitamin D.

	Sample size	Age in years	Diagnosis	Study design	Outcome
Nwosu et al., 2017 (95)	171	16.5 ± 3.1	IBS diagnosed by ROME III criteria	Retrospective case-control	Patients with IBS had significantly lower 25-hydroxy-vitamin D concentration compared to controls
El Amrousy et al. (101)	112	14–18	IBS with Vitamin D deficiency	Randomized controlled trial	IBS patients who received Vitamin D supplementation showed significant improvement in IBS symptoms, quality of life compared to placebo group

## Electrical stimulation

### Gastric electrical stimulation (GES) and percutaneous electrical nerve field stimulation (PENFS)

GES and PENFs have been used to treat pediatric FGIDs. The mechanism of action of GES is not clear. Electrical simulation therapies have not been shown to stimulate gastric muscle contraction or contribute to pacing or propagation (entrainment) of gastric slow waves regulated by the interstitial cells of cajal (103). Other therapeutic mechanisms of action proposed include improving gastric accommodation and modulating vagal tone (104). GES has been used in the past few years as a treatment option for children with gastroparesis, chronic nausea and FD. A retrospective 2016 study by Islam et al. studied 96 patients who underwent temporary GES and 67 patients who had a permanent GES device surgically implanted (105). Patients in the study ranged from 2 to 19 years of age and most patients (69.1%) had other comorbidities with autoimmune disease (17.5%) and genetic/congenital issues (24.7%) being the 2 most common (105). There was significant symptom improvement in 68.75% of patients (66/96) who underwent temporary GES (105). 98.5% (66/67) of patients who underwent permanent GES implantation reported reduction in all symptoms with 90% having a sustained response >1 year (105). PENFS targets specific pain areas in the central nervous system (i.e., the amygdala) through stimulation of auricular branches of nerves supplying the gut (106). A RCT that enrolled 115 adolescents showed patients treated with PENFS had significant improvement in pain scores, global well-being and functional disability compared to the placebo group as shown in Table 12 (107). A recent prospective single arm trial conducted by Santucci et al. enrolled 20 children with FGIDs and found PENFs treatment resulted in improvements in resting and evoked pain, nausea, sleep, disability, catastrophizing, somatic complaints and anxiety after 4 weeks of therapy with sustained results 6–12 months post treatment (108).

**Table 12:** Gastric electrical stimulation (GES) and percutaneous electrical nerve field stimulation (PENFS).

	Sample size	Age in years	Diagnosis	Study design	Outcome
Islam et al., 2016 (105)	96	2–19	Gastroparesis	Retrospective observational	Significant improvement in symptoms in patients who underwent temporary as well as permanent GES implantation at 1,6 and ≥12 months
Kovacic et al., 2017 (107)	115	11–18	FGID diagnosed by ROME III criteria	Double blind RCT	Subjects in the PENFS group had greater reduction in worst pain compared to placebo at 3 weeks of treatment
Santucci et al., 2022 (108)	20	11–19	FD, IBS, FAP-NOS diagnosed based on ROME IV criteria	Prospective non blinded clinical trial	PENFs treatment resulted in improvements in resting and evoked pain, nausea, sleep, disability, catastrophizing, somatic complaints and anxiety after 4 weeks of therapy with sustained results 6–12 months post treatment

## Conclusion

This review highlights non-pharmacologic therapies clinicians may offer as treatment options for pediatric patients with IBS and reviews the relevant literature. It also highlights some common non-pharmacologic therapies patients may inquire about but may not be recommended due to lack of supporting evidence. In general, well conducted pediatric trials for many therapies discussed are lacking and the existing ones are small in sample size making generalizable conclusions difficult. Treatments offered to patients should consider the available evidence, the individual patient circumstances, and resource availability.
